# First Global Images of Ion Energization in the Terrestrial Foreshock by the Interstellar Boundary Explorer

**DOI:** 10.1029/2020GL088188

**Published:** 2020-08-12

**Authors:** M. A. Dayeh, J. R. Szalay, K. Ogasawara, S. A. Fuselier, D. J. McComas, H. O. Funsten, S. M. Petrinec, N. A. Schwadron, E. J. Zirnstein

**Affiliations:** ^1^ Space Science and Engineering Division Southwest Research Institute San Antonio TX USA; ^2^ Department of Physics and Astronomy University of Texas at San Antonio San Antonio TX USA; ^3^ Department of Astrophysical Sciences Princeton University Princeton NJ USA; ^4^ ISR Division Los Alamos National Laboratory Los Alamos NM USA; ^5^ Lockheed Martin Advanced Technology Center Palo Alto CA USA; ^6^ Space Science Center University of New Hampshire Durham NH USA

**Keywords:** energetic neutral atoms, charge‐exchange, ion acceleration

## Abstract

The Interstellar Boundary Explorer (IBEX) mission provides global energetic neutral atom (ENA) observations from the heliosphere and the Earth's magnetosphere, including spatial, temporal, and energy information. IBEX views the magnetosphere from the sides and almost always perpendicular to noon‐midnight plane. We report the first ENA images of the energization process in the Earth's ion foreshock and magnetosheath regions. We show ENA flux and spectral images of the dayside magnetosphere with significant energization of ENA plasma sources (above ~2.7 keV) in the region magnetically connected to the Earth's bow shock (BS) in its quasi‐parallel configuration of the interplanetary magnetic field (IMF). We also show that the ion energization increases gradually with decreasing IMF‐BS angle, suggesting more efficient suprathermal ion acceleration deeper in the quasi‐parallel foreshock.

## Key Points

- We present the first remote‐sensing global images of ion energization in the Earth's foreshock
- We provide ion energization profile as a function of bow shock obliquity
- We quantify the difference of energization in magnetosheath and its magnetically connected counterpart in the upstream foreshock

## 1 Introduction

### 1.1 The Earth's Foreshock

The Earth's foreshock is a dynamic region upstream of the bow shock (BS). In the foreshock, solar wind (SW) magnetic field lines are magnetically connected to the BS. The properties of the foreshock are governed by the interaction of three main parameters: interplanetary magnetic field (IMF) direction, BS normal, and SW flow vector. Noncoplanarity of these three components defines the 3‐D structure of the foreshock with large spatial morphology. Origins of plasma populations in the foreshock are debatable. Sources include leakage of downstream heated ions (e.g., Edmiston et al., 1982) or reflected SW ions at the BS (e.g., Fuselier, 1995). The resulting complex accelerating processes and plasma populations make it nearly impossible to individually examine these processes separately with single‐spacecraft in situ measurements.

**Figure 1:**
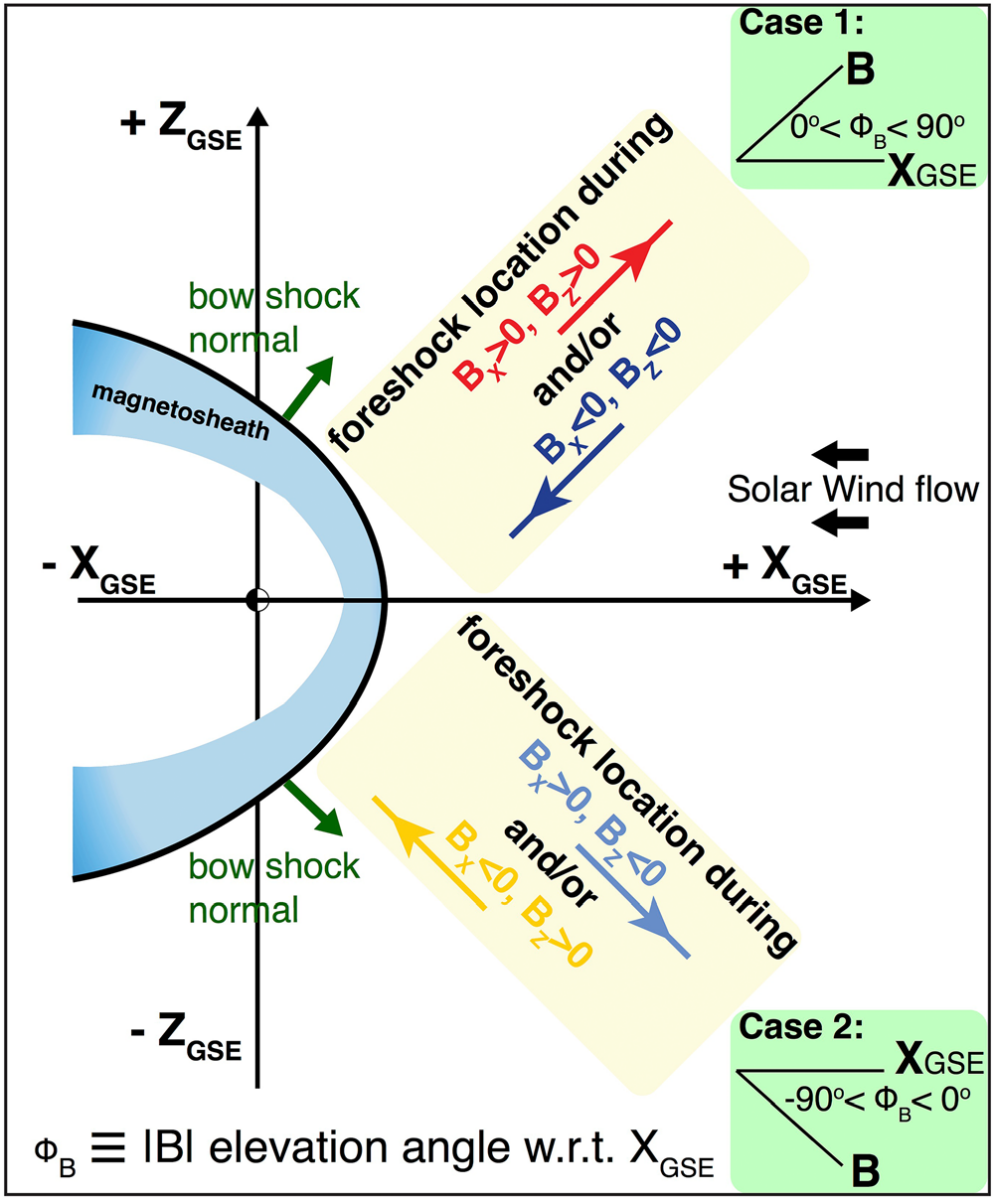
Locations (Cases 1 and 2) of the Earth foreshock region when the IMF B_y_ component is small as viewed in the XZ_GSE_ plane. Shown are two configurations of IMF dominated by its B_x_ and B_z_ components, which result in two configurations of the quasi‐parallel bow shock.

Nevertheless, given its 3‐D structure upstream of the BS, the foreshock can be projected in 2‐D, in the X_GSE_Y_GSE_ or X_GSE_Z_GSE_ planes, depending on the IMF B_z_ configuration, which is the key quantity for most variations among the three parameters described above. We define the elevation angle, ∅_*B*_, to be the angle between the IMF direction and the X_GSE_, the direction from Earth to the Sun within the X_GSE_Z_GSE_ plane. Figure 1 illustrates two cases in which the foreshock can be observed using remote sensing from the SW outside the BS on the dawn or dusk flanks. For Case 1 (0°< ∅_*B*_ < 90°), the foreshock is observed in the [+X_GSE_, +Z_GSE_] hemisphere under the following conditions: [B_x_ > 0, B_z_ > 0] or [B_x_ < 0, B_z_ < 0]. In contrast, for Case 2 (−90°< ∅_*B*_ < 0°), the foreshock is in the [+X_GSE_, −Z_GSE_] hemisphere under the conditions [B_x_ > 0, B_z_ < 0] or [B_x_ < 0, B_z_ > 0].

Measurements from ISEE‐1 and ISEE‐2 (in the 1970s and 1980s) laid the foundation for understanding the foreshock region's ion populations and structure (e.g., Bonifazi et al., 1983). Observations revealed that the foreshock region is comprised of a complex mixture of suprathermal‐through‐energetic (~1 to >100 keV) particles along with intense wave‐particle interactions. The mechanisms that create these ion populations are debated (Burgess et al., 2012; Eastwood et al., 2005; Fuselier, 1995; and references therein). The following main populations and production processes decrease monotonically with shock obliquity angle, $\theta_{B_{n}}$, defined as the angle between the IMF and the normal to the BS surface:

- *Field‐aligned beams*: high‐energy (>10 keV), field‐aligned beams at the upstream edge (near $\theta_{B_{n}}$~45°) produced by shock drift acceleration of a small portion of the incident SW ions.
- *Intermediate distributions*: low‐energy (~1 keV), field‐aligned beams produced by SW ions reflected from the BS as well as heated SW ions leaked from the foreshock and magnetosheath regions. These reflected particles create low‐frequency waves that trap the beams into gyrophase‐bunched populations. The particles later pitch angle scatter and form “intermediate distributions” that are distinguishable from the field‐aligned beams.
- *Diffuse distributions*: very high‐energy (>100 keV) particles accelerated deep in the foreshock. These appear to be fully scattered and developed distributions with other potential sources within the foreshock. However, the high‐energy tail of this population results mostly from the efficient first‐order, Fermi‐acceleration mechanism in the vicinity of the turbulent quasi‐parallel (*Q*_∥_) BS region.

A full review of the foreshock distributions and processes, partially described above, can be found in Burgess (1997), Eastwood et al. (2005), Fuselier (1995), and Greenstadt et al. (1995).

### 1.2 Global Perspective of the Foreshock Region

Studying the global processes in the foreshock region requires simultaneous measurements of plasma properties in the magnetosheath and the upstream SW. While this is not possible with single‐point in situ spacecraft observations, statistical studies based on long‐term surveys have enabled the development of a qualitative understanding of this region. These results generally pertain to the properties of thermal plasma populations, bulk flows, and spatial asymmetries (e.g., Dimmock & Nykyri, 2013; Lavraud et al., 2013; Longmore et al., 2005; Némećek et al., 2000; Paularena et al., 2001; Walsh et al., 2012; and references therein). Nevertheless, it has been difficult to establish a quantitative relation between the local ion populations and the upstream SW conditions for two main reasons. First, in situ spacecraft sample highly localized regions at any specific time, thus spatial and temporal variations of the observations critical to resolve physical processes cannot be uniquely distinguished. Second, in situ observations for single spacecraft do not provide information about the processes potentially acting near the sampled region, such as BS motion and transient events. While multispacecraft formations (e.g., Cluster, THEMIS, and MMS) are used to examine small‐scale processes, remote measurements further provide a broader image of these processes on very large spatial scales.

A fraction of the foreshock ions charge‐exchange with the cold exospheric neutral atoms in the Earth's geocorona, creating energetic neutral atoms (ENAs; Fuselier et al., 2010; McComas et al., 2011). Newly created ENAs propagate along the gyromotion direction at the time of the charge exchange, maintaining the original energy of the progenitor ions. At a certain fixed point in space, these ENAs can be observed remotely, enabling global imaging of this region. Similar ENA imaging has been exploited extensively to understand the physical processes in different parts of the Earth's magnetosphere (e.g., Dayeh et al., 2015; McComas et al., 2011; Ogasawara et al., 2015, 2019). Furthermore, ENA remote sensing continuously images the region of interest over multiple spatial scales, hence simultaneously viewing emissions from different portions of the region of interest (Dayeh et al., 2015; Ogasawara et al., 2019). This enables us to directly relate the imaged region's properties to the upstream SW and IMF conditions.

In this paper, we utilize the unique orbital configuration of the Interstellar Boundary Explorer (IBEX; McComas et al., 2009) mission and the high sensitivity of its ENA imagers to construct the first global 2‐D images of the dayside region, revealing the energization effects in the terrestrial foreshock region. We present flux and spectral images of the particle populations showing significant energization of ENA plasma sources in the foreshock location for two IMF configurations. We also show ion energization profiles in the magnetosheath and foreshock and discuss their similarities and differences as a function of shock obliquity. Finally, we show that ion energization increases further back along the shock as it becomes more *Q*_∥_, suggesting more efficient suprathermal ion acceleration deeper in the *Q*_∥_ foreshock, possibly by diffusive shock‐acceleration processes.

## 2 Data Sets and Selection Criteria

We use hydrogen ENA observations from the IBEX mission, which launched in October 2008 to image the source H^+^ plasma populations involved in the interaction between the SW and the interstellar medium. It is equipped with two single‐pixel (conical 6.5° FWHM field‐of‐view) ENA cameras, IBEX‐Lo (Fuselier et al., 2009) and IBEX‐Hi (Funsten et al., 2009), which cover an overlapping energy range from 0.01 to 2 keV and 0.5 to 6 keV, respectively. The IBEX spacecraft is nominally sun‐pointing, and the field‐of‐view of each imager is perpendicular to the spin axis. Over each ~14.5 s spin, ENAs are detected continuously from a great circle (6.5° × 360°) projected onto the sky (direct events) and are also binned onboard every 96 spins (~23 min).

**Figure 2:**
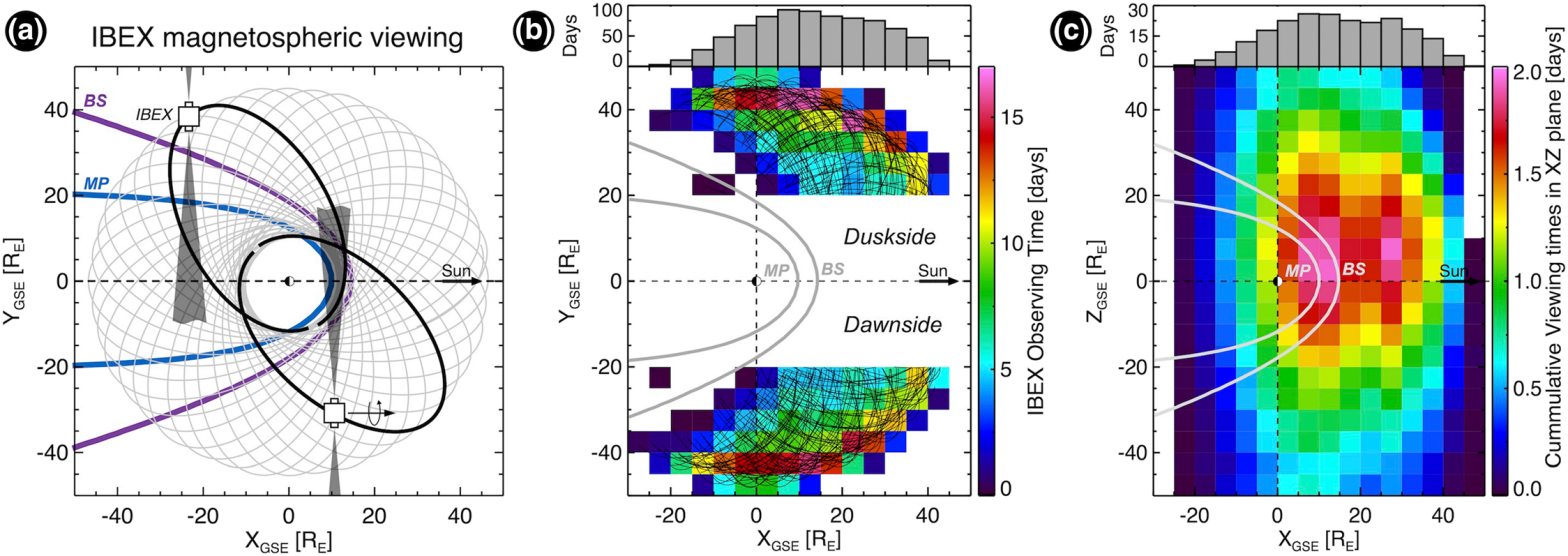
(a) Orbital configurations of IBEX during 2009, illustrating its viewing of the distant magnetospheric regions for a significant amount of time. (b) Cumulative time of IBEX location in orbit in the XY plane during all orbits used in this study. (c) Cumulative viewing time across the entire XZ plane during the study period, as viewed by the IBEX‐Hi instrument.

IBEX was inserted into a highly elliptical orbit at low inclination (McComas et al., 2009; Scherrer et al., 2009) and thus regularly observes the magnetosphere from the dawn and dusk sides. From these vantage points, IBEX acquires slices of the magnetosphere as it moves along its orbit. Composite images are then constructed over different time periods for specified SW conditions (e.g., Dayeh et al., 2015; Fuselier et al., 2010; McComas et al., 2011; Ogasawara et al., 2013; Petrinec et al., 2010). This combination of orbital viewing and sensitive ENA cameras form an excellent platform to observe ENA emissions from particle populations in the Earth's magnetosphere and adjacent shocked SW environments for long periods of time and from distances up to ~40 Earth radii. Ideal magnetospheric viewing periods occur up to 8 months per year (between September–December and April–July). Figure 2a shows the orbital configurations of IBEX during 2009, illustrating its magnetospheric viewing. Figure 2b shows the total time (in days) for which IBEX views the magnetosphere from different locations in orbit, and Figure 2c shows the cumulative viewing time across the X_GSE_Z_GSE_ plane. ENAs measured by IBEX represent the combined emissions of potentially multiple ion populations along an individual line of sight, although ion populations that are comparatively abundant and/or have sharp spatial boundaries tend to dominate the features and spectra within acquired ENA images. For this study, we use the ENA differential fluxes ( *j*_ENA_) calculated from the IBEX‐Hi direct‐event histogram data binned at 6° resolution. The data covers orbits 18 through 431 between 2009 and 2018 at two energy passbands centered at *E*_1_ = 2.7 keV and *E*_2_ = 4.3 keV. Each of the 6° bins is then projected into the noon‐midnight plane. All projected pixels are then statistically averaged across the XZ plane, forming a 2‐D projection of measured ENAs.

ENA emission is produced by energy‐dependent charge exchange of plasma ions with cold, ambient geocoronal neutral atoms. Our goal is to understand the source ion populations and compare their line‐of‐sight densities at different energies, so we derive a proxy line‐of‐sight ENA flux *j*_i_ (*E*), that is proportional to the ion flux, by removing the energy dependence of the H^+^ + H^0^ → H^0^ + H^+^ charge exchange cross section σ_HH_(*E*) (Lindsay & Stebbings, 2005) according to

(1) $$j_{i}\left(E\right)=j_{\mathrm{ENA}}\left(E\right)/\sigma_{\mathrm{HH}}\left(E\right).$$

Note that ENA fluxes measured by IBEX do not simply indicate those of the source ions. The latter requires knowing both the ion distribution along IBEX line‐of‐sight and the geocoronal neutral density as a function of position (longitude, latitude) from Earth (e.g., Rairden et al., 1986; Zoennchen et al., 2010). Incorporating this detailed 3‐D density profile model for the geocorona is beyond the scope of this observational paper. However, the analysis here focuses on the global profile of spectral indices and not on the absolute ENA fluxes; the former is independent of the geocoronal density and therefore reflects the source ion properties.

SW and IMF parameters are from the 1‐min OMNI data set convected to the Earth's subsolar point. We use OMNI SW data in setting the selection constraints on the IBEX ENA data. Note that OMNI cadence is 1 min while IBEX binned data are averaged over ~23 min. We first average the OMNI data over 23 min to match the time cadence of IBEX data. The selection criteria indicated earlier then filter out all data gaps and IBEX data that do not meet the SW‐set conditions.

## 3 Observations

### 3.1 Imaging Ion Energization at the Foreshock

Using IBEX‐Hi data in the 2.7 and 4.3 keV passbands, we impose the following conditions to select conditional data and construct the foreshock image for two different IMF directions, as follows.

Case 1: where ∅_*B*_ is positive, and the foreshock is expected to be primarily in the Northern Hemisphere (Figure 1) under the constraints

(2) $$\text{Case}\;1:\left\{\begin{array}{l} \left|Y_{\mathrm{GSE}}\right|>20\;R_{E}\\ {\mathrm{Bz}}_{\mathrm{GSE}}>2.0\;\mathrm{nT}\\ {\mathrm{Bx}}_{\mathrm{GSE}}>2.0\;\mathrm{nT}\\ \left|\frac{{\mathrm{Bz}}_{\mathrm{GSE}}}{{\mathrm{By}}_{\mathrm{GSE}}}\right|>1.0 \end{array}\right)\;\text{or}\;\left\{\begin{array}{l} \left|Y_{\mathrm{GSE}}\right|>20\;R_{E}\\ {\mathrm{Bz}}_{\mathrm{GSE}}<‐2.0\;\mathrm{nT}\\ {\mathrm{Bx}}_{\mathrm{GSE}}<‐2.0\;\mathrm{nT}\\ \left|\frac{{\mathrm{Bz}}_{\mathrm{GSE}}}{{\mathrm{By}}_{\mathrm{GSE}}}\right|>1.0 \end{array}\right).$$

Case 2, where ∅_*B*_ is negative, and the foreshock is expected to be primarily in the Southern Hemisphere (Figure 1) under the constraints:

(3) $$\text{Case}\;2:\left\{\begin{array}{l} \left|Y_{\mathrm{GSE}}\right|>20\;R_{E}\\ {\mathrm{Bz}}_{\mathrm{GSE}}>2.0\;\mathrm{nT}\\ {\mathrm{Bx}}_{\mathrm{GSE}}<‐2.0\;\mathrm{nT}\\ \left|\frac{{\mathrm{Bz}}_{\mathrm{GSE}}}{{\mathrm{By}}_{\mathrm{GSE}}}\right|>1.0 \end{array}\right)\;\text{or}\;\left\{\begin{array}{l} \left|Y_{\mathrm{GSE}}\right|>20\;R_{E}\\ {\mathrm{Bz}}_{\mathrm{GSE}}<‐2.0\;\mathrm{nT}\\ {\mathrm{Bx}}_{\mathrm{GSE}}>2.0\;\mathrm{nT}\\ \left|\frac{{\mathrm{Bz}}_{\mathrm{GSE}}}{{\mathrm{By}}_{\mathrm{GSE}}}\right|>1.0 \end{array}\right).$$

In both Cases 1 and 2, the condition ∣Y_GSE_ ∣ >  20 R_E_ ensures that IBEX is always outside the BS, and the condition $\left|\frac{{\mathrm{Bz}}_{\mathrm{GSE}}}{{\mathrm{By}}_{\mathrm{GSE}}}\right|$ >1.0 ensures that the IMF B_z_ component is always larger than B_y_.

**Figure 3:**
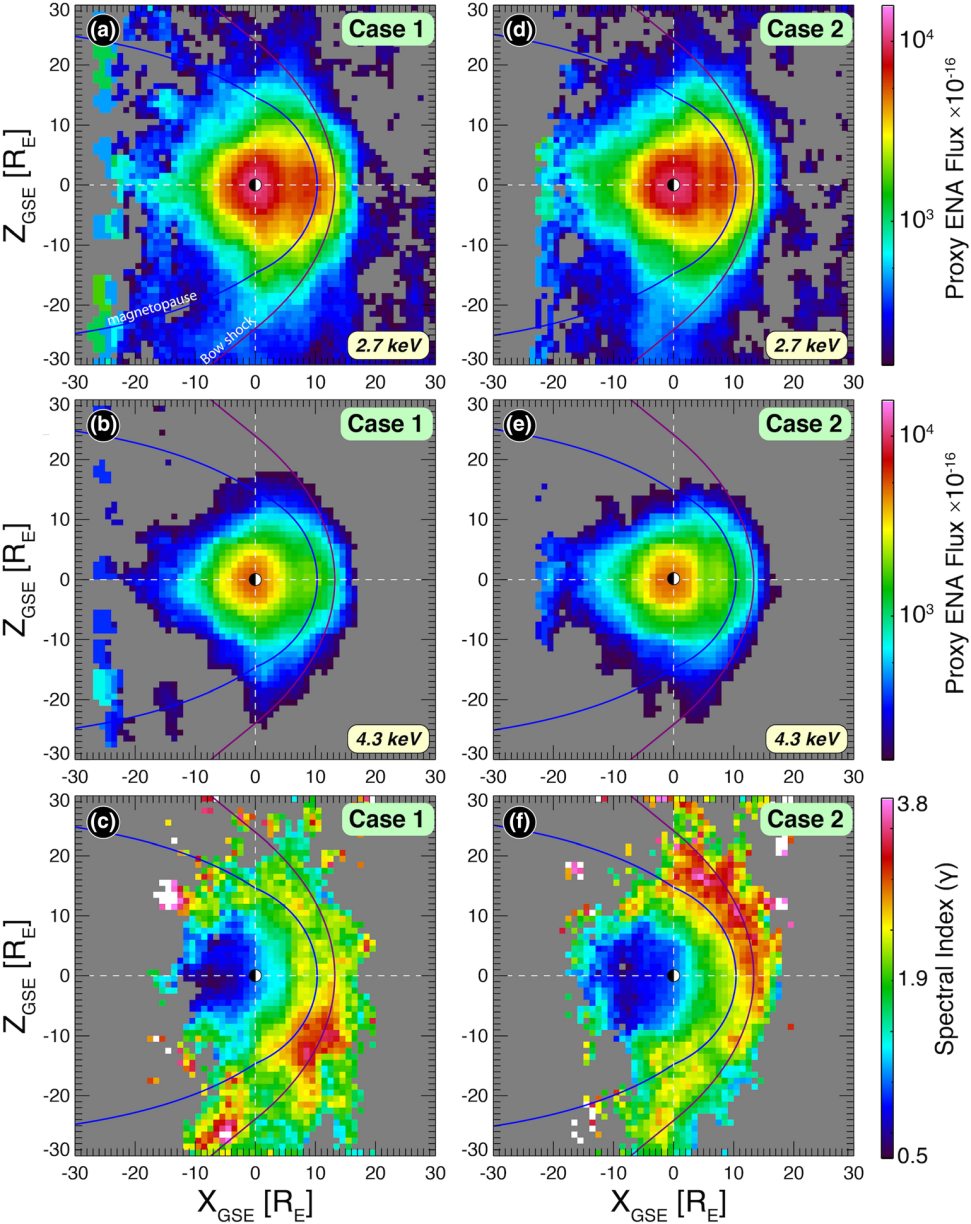
(a, b) Proxy ENA fluxes derived from IBEX ENA fluxes and projected onto the XZ plane are shown for 2.7 and 4.3 keV for Case 1. Shown fluxes are scaled by multiplying by 10^−16^. (c) Spectral index *γ* plot using the proxy ENA fluxes are shown in panels (a) and (b). The *Q*_∥_ bow shock region is where the spectrum is harder (smaller index). The softer spectra (larger index) represent the *Q*_⊥_ region of the bow shock where particles do not accelerate to higher energies. (d–f) Similar to panels (a)–(c) but for Case 2. In both panels (c) and (f), there is no constraint on the elevation angle, |∅_*B*_|, which varied within 0° to 90° in each hemisphere.

The resulting images are shown in Figure 3. Figures 3a and 3b show the proxy ENA fluxes at 2.7 and 4.3 keV, respectively. As expected, ENA fluxes are higher closer to Earth, owing to the exospheric geocoronal density profile. The averaged curvature of the magnetosphere boundary is also apparent. Superposed traces are the averaged BS and magnetopause (purple and blue, respectively) determined from the averaged upstream SW conditions convected to the subsolar point (Chao et al., 2002; Shue et al., 1998). Figure 3c shows the spectral index image determined from Figures 3a and 3b, using

(4) γ=log10j1E1j2E2log10E1E2,

where *γ* is the spectral index and *j*_1_(*E*_1_) and *j*_2_(*E*_2_) are the proxy ENA fluxes at 2.7 and 4.3 keV, respectively (see Equation 1).

Under a positive ∅_*B*_ configuration (Case 1), the Northern Hemisphere [+X_GSE_, +Z_GSE_] is the *Q*_∥_ region of the BS, and the Southern Hemisphere [+X_GSE_, −Z_GSE_] is quasi‐perpendicular (*Q*_⊥_). The anticipated foreshock is thus located primarily in the Northern Hemisphere, as shown in Figure 3c. The fact that the latter is characterized by a harder (i.e., smaller) spectral index than the southern one suggests that the higher‐energy component (4.3 keV) is less pronounced in the southern *Q*_⊥_ region, indicating minimal acceleration in this region.

Figures 3d–3f have the same format as Figures 3a–3c, but these figures are for Case 2, where ∅_*B*_ < 0°, thus the *Q*_∥_ and *Q*_⊥_ regions are opposite to those of Case 1. Indeed, the foreshock in this case appears predominantly in the Southern Hemisphere, as expected. The global features are similar, mainly characterized with a hard spectrum in the foreshock region indicating energization of the high‐energy parent ion component.

### 3.2 Sensitivity to Variations in the IMF Elevation Angle, ∅_*B*_

In Figure 3, we inferred the foreshock energization under a positive IMF elevation angle (∅_*B*_ > 0°, Case 1) and a negative IMF elevation angle (∅_*B*_ < 0°, Case 2), where each ∣∅_*B*_∣ varied within 0° to 90° along the entire perspective hemisphere. Next, we add an additional constraint to Equations 2 and 3 so that we test the sensitivity of the foreshock global detection to variations in the elevation angle.

To do so, we first determine the histogram of ∅_*B*_ values for all data used (Figure S1a in the supporting information, Case 1); we then select a subset of the data within a specific range of ∅_*B*_ to reconstruct the foreshock image. Figures S1b and S1c show the resulting images using selection window ranges of 30–60° and 25–55°, respectively, around a nominal ∅_*B*_ = 45° angle. Figures S1d–S1f are similar but for Case 2.

As shown in both cases, structural variations exist in the spectral map. However, the foreshock signature and presence stand out, confirming that varying the IMF elevation angle within each hemisphere does not largely affect the findings.

### 3.3 Energization as a Function of Shock Obliquity

**Figure 4:**
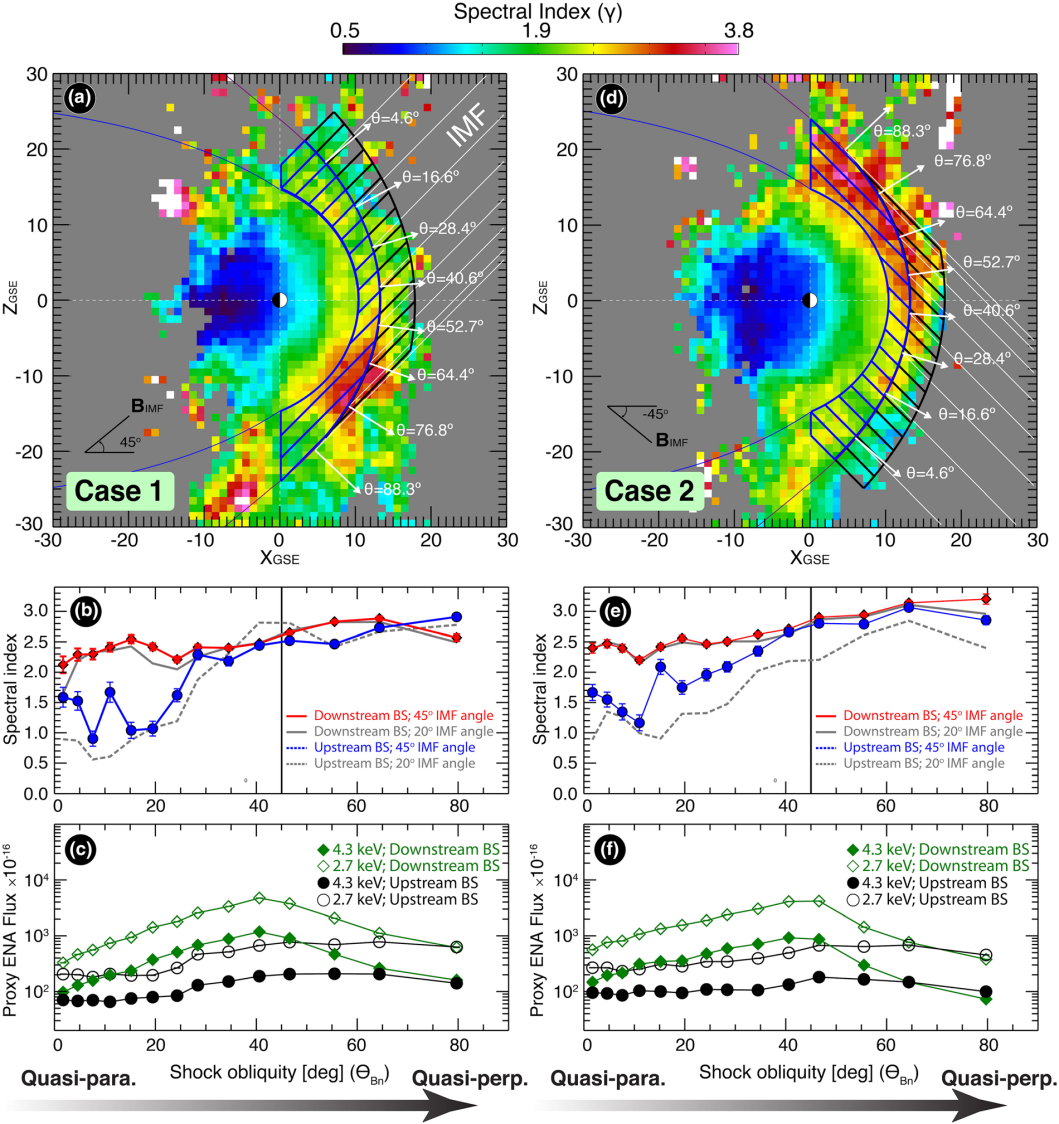
(a) Sectored masks covering most of the magnetosheath and the foreshock regions. The masks are aligned with an IMF ∅_*B*_ of 45° and are ~3° wide. (b) Variations of the spectral indices in the masking sectors as a function of shock obliquity in the magnetosheath (red) and the foreshock (blue) regions. Gray curves represent the values derived using the 20° constraint on $\theta_{B_{\mathrm{xz}}}$. (c) Proxy ENA fluxes at 2.7 and 4.3 keV in the masking sectors of the indicated magnetosheath and foreshock regions. Panels (d)–(f) have the same format of panels (a)–(c) but for Case 2.

Next we investigate how ion energization changes as a function of shock obliquity angle, $\theta_{B_{n}}$, as it varies from the *Q*_⊥_ to the *Q*_∥_ configuration at the BS. We first create a sectored mask (each with 14 sectors covering $1{}^{\circ}<\theta_{B_{n}}<80{}^{\circ}$) for the foreshock (black sectors in Figure 4) and magnetosheath (blue sectors in Figure 4) regions. The boundary between the upstream and downstream masks represents the location of the BS as determined from the averaged upstream conditions using the BS model from Chao et al. (2002). Sectored masks along the Z direction are ~3 RE wide. Figure 4a shows the foreshock image constrained at 0°< ∅_*B*_ < 90°. We superposed sectored masks covering the downstream and upstream regions, aligned along the same IMF ∅_*B*_. Figure 4b shows the weighted‐average of the spectral indices in each mask sector downstream (red trace) and upstream of the BS (blue trace), Gray traces show results from the 20° window to examine the sensitivity of the spectral trend to IMF elevation angle changes.

Figures 4b and 4e reveal several important features: (1) The spectral index, *γ*, in the *Q*_∥_ region is always smaller than that of the *Q*_⊥_ region. (2) *γ* increases monotonically from *Q*_∥_ to the *Q*_⊥_. This increase in *γ* corresponds to a decreasing rate of energization, moving away from the deeper foreshock. (3) On average, the monotonic trend in *γ* from *Q*_∥_ to *Q*_⊥_ region is gradual. (4) At low shock obliquity and deep in the foreshock region ( $\theta_{B_{n}}<20{}^{\circ}$), the spectral index downstream of the BS (in red) is significantly larger than its upstream counterpart (in blue). We note that ion spectra in the foreshock vary under different shock conditions and could change drastically on a scale of minutes (e.g., Meziane et al., 2004; Scholer et al., 1990). Because of such fast‐acting dynamics and the complicated combination of processes, these spectral indices averaged over long times and with limited energy resolution ought to be interpreted from a global perspective only. Nonetheless, they could act as a measure of the energetic particle production compared to the cold source populations in the local region.

Figures 4c and 4d show the averaged proxy ENA fluxes in both regions determined at 2.7 and 4.3 keV. Fluxes appear to exhibit an asymmetry (more clear in Figure 4f) with a skew toward the *Q*_∥_ region, likely caused by the accelerated population in this region. The consistent behavior of a hard spectrum in the *Q*_∥_ region of the shock and a softer spectrum in the *Q*_⊥_ region of the shock strongly suggest that the *Q*_∥_ portion of the shock is where the ion energization takes place and decreases gradually with increasing $\theta_{B_{n}}$.

## 4 Discussion and Conclusions

This paper presents the first‐ever global images of ion energization in the Earth's foreshock, obtained from remote sensing. We find a clear relation between the spectral index of the source ion populations and shock obliquity, which varies along the entire dayside region of the magnetosphere. We emphasize that this image is inherently different from global images constructed overtime from a large set of single‐point in situ observations. In the former, there is almost simultaneous imaging of adjacent pixels over similar global conditions, whereas in the in situ case, observations of adjacent regions can be very long. This study is thus the first that enables examining adjacent regions upstream and downstream of the BS over the entire dayside.

Our results are summarized in the following key points:

1. Energization due to acceleration processes in the magnetosheath and the foreshock becomes more efficient with decreasing shock obliquity (as the shock normal becomes more parallel to the IMF). When averaging the magnetosheath and upstream BS regions for both cases described in this study (Figures 4b and 4e), the weighted average of the spectral index increases from 1.93 ± 0.03 in the *Q*_∥_ case ( $\theta_{B_{n}}<45{}^{\circ}$) to 2.63 ± 0.02 in the *Q*_⊥_ case ( $\theta_{B_{n}}>45{}^{\circ}$).
2. Energization upstream of the BS is stronger than the downstream region that is magnetically connected to it, especially where $\theta_{B_{n}}<30{}^{\circ}$. While this could be due to a more efficient acceleration, it could also be due to a transport effect where the lower energy fraction does not easily move along the field lines connected to the upstream region.

ENA images also show that the energization along the BS fluctuates during the different presented conditions, sometimes extending beyond the sub solar point or falling short of it. These statistical fluctuations are a natural consequence of the long time averages used in constructing the images.

These results also agree with key aspects of previous studies. Ogasawara et al. (2015) performed a statistical study of suprathermal ions as a function of shock obliquity in the subsolar magnetopause. They used IBEX data, finding that suprathermal ion acceleration was more efficient at the *Q*_∥_ region of the downstream bowshock near the subsolar point (focus point of their analysis). The authors suggested that shock acceleration processes rather than magnetic reconnection near the magnetopause dominate magnetosheath heating. Furthermore, observations from ISEE‐1 confirm that the existence of >3 keV particles are a function of shock obliquity (Crooker et al., 1981). Crooker et al. (1981) attributed the presence of these energetic ions to the efficient diffusive shock acceleration of ions in the *Q*_∥_ region of the BS. Our results support these previous findings and expand on their limited measurements to cover the entire regions upstream and downstream of the BS, showing that similar acceleration process operate on a global scale around the BS. These findings provide observational constraints on the thermodynamic processes in this region.

While the foreshock location is very clear in IBEX's ENA images, we note that it is not possible for IBEX to identify the details of the various acceleration processes operating in the foreshock. This is mainly due to the long‐exposure acquisitions and the long‐term averaging of the data. Nevertheless, the results identify the range of energies involved in the process as a function of obliquity, as well as the spatial region of operations. Furthermore, ENA images presented here are created from ions that are accelerated via different processes in the foreshock and magnetosheath, including diffusive shock acceleration, drift shock acceleration, and wave‐particle interactions (e.g., Fuselier, 1995). Thus, we have provided the first global images of ion energization in the foreshock obtained by remote sensing. We have also shown how energization varies across the shock as a function of its obliquity, along with qualitative differences of ion energization upstream and downstream of the BS. This work demonstrates the importance of ENA imaging in capturing the global dynamics of the SW interactions with the magnetosphere and sets the ground for future missions that utilize ENA imaging at finer spatial and temporal resolutions.

## Supporting information

Figure S1

## Data Availability

IBEX observations are available through the official IBEX release website, https://ibex.princeton.edu/DataRelease12, as well as by contacting the IBEX PI, Prof. D. J. McComas at the Princeton University (dmccomas@princeton.edu). OMNI data were obtained from the GSFC/SPDF OMNIWeb interface at http://omniweb.gsfc.nasa.gov, which are derived from multi‐spacecraft SW plasma and magnetic field observations.
