# Supplementary material for: First Global Images of Ion Energization in the Terrestrial Foreshock by the Interstellar Boundary Explorer
Source: Geophys Res Lett. 2020 Aug 12;47(16):e2020GL088188. doi: 10.1029/2020GL088188 (PMC7583366; doi:10.1029/2020GL088188)
Supplement: Supplementary file 1 — Figure S1 [file GRL-47-e2020GL088188-s001.docx]

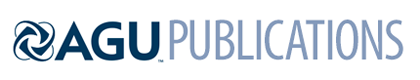


*[Geophysical Research Letters]*

Supporting Information for

**[First global images of ion energization in the terrestrial foreshock by the Interstellar Boundary Explorer]**

M. A. Dayeh^1,2^ , J. R. Szalay^3^, K. Ogasawara^1^, S. A. Fuselier^1,2^, D. J. McComas^3^, H. O. Funsten^4^, S. M. Petrinec^5^, N. A. Schwadron^6^, and E. J. Zirnstein^3^

*^1^ Southwest Research Institute, San Antonio, TX 78238 (*maldayeh@swri.*edu)*

*^2^ University of Texas at San Antonio, San Antonio, TX 78249*

*^3^ Princeton University, Princeton, NJ, 08544*

*^4^ Los Alamos National Laboratory, Los Alamos, NM 87545*

*^5^ Lockheed Martin Advanced Technology Center, Palo Alto, CA 94304*

*^6^ University of New Hampshire, Durham NH 03824*

**Contents of this file**

Figure S1

**Supporting figure for §3.2, Sensitivity to variations in the IMF elevation angle** $\boldsymbol{\emptyset}_{\boldsymbol{B}}$

We determine the histogram of $\boldsymbol{\emptyset}_{\boldsymbol{B}}$ values for all data used (Figure S1a, Case 1) and select a subset of the data within a specific range of $\boldsymbol{\emptyset}_{\boldsymbol{B}}$ to reconstruct the foreshock image. Figures S1b and S1c show the resulting images using selection window ranges of 30^o^ - 60^o^ and 25^o^ - 55^o^, respectively, around a nominal $\boldsymbol{\emptyset}_{\boldsymbol{B}}\boldsymbol{=45^{\circ}}$ angle. Figures S1d, S1e, S1f are similar, but for Case 2. As shown in both cases, structural variations exist in the spectral map. However, the foreshock signature and presence stand out, confirming that varying the IMF angle within each hemisphere does not largely affect the findings.

***
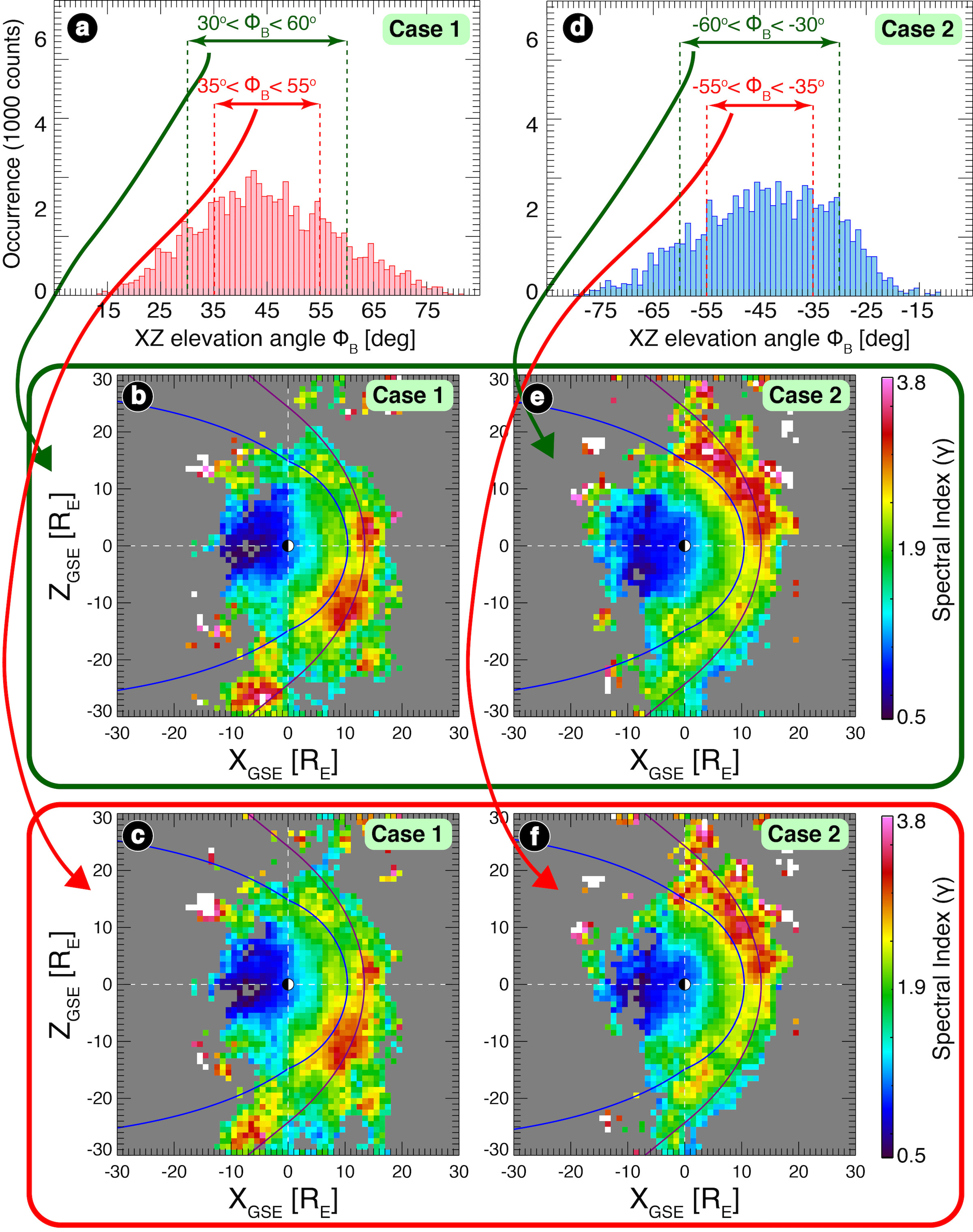
***

***Figure S1.*** *(a) Histogram of* $\boldsymbol{\emptyset}_{\boldsymbol{B}}$ *during the studied period for Case 1, examining two band selections centered around 45^o^. (b,c) Spectral images similar to Figure 3d, but using two constraints on the elevation angle* $\boldsymbol{\emptyset}_{\boldsymbol{B}}$ *as illustrated in (a). (d,e,f) Similar to (a,b,c) but for Case 2.*
